# AlScN Thin Films for the Piezoelectric Transduction of Suspended Microchannel Resonators

**DOI:** 10.3390/s25175370

**Published:** 2025-08-31

**Authors:** Yara Abdelaal, Marco Liffredo, Luis Guillermo Villanueva

**Affiliations:** Advanced NEMS Laboratory, École Polytechnique Fédérale de Lausanne (EPFL), Route Cantonale, 1015 Lausanne, Switzerland; marco.liffredo@epfl.ch (M.L.); guillermo.villanueva@epfl.ch (L.G.V.)

**Keywords:** suspended microchannel resonator, piezoelectric transduction, thin film characterization

## Abstract

**Highlights:**

**What are the main findings?**

- Demonstration of a fourfold increase in the d_31_ piezoelectric coefficient Al_0.6_Sc_0.4_N films compared to AlN.
- Examination of the influence of bottom electrode patterning design, photoresist removal, and deposition bias on film quality and transduction performance.

**What is the implication of the main finding?**

- Establishing Al_0.6_Sc_0.4_N as a promising material for high-performance, fully piezoelectrically transduced suspended microchannel resonators (SMRs).

**Abstract:**

Suspended microchannel resonators (SMRs) are powerful tools for mass, density, and viscosity sensing. Among various transduction methods, full piezoelectric transduction offers key advantages, including on-chip integration, low energy dissipation, and linear response. This work explores sub-200 nm Al_0.6_Sc_0.4_N thin films for SMR transduction, benchmarking them against their well-established AlN predecessor. By integrating the piezoelectric stack into low-stress silicon nitride (ls-SiN_x_) beam resonators, we investigate the impact of bottom electrode design, photoresist removal prior to deposition, and deposition bias on film quality. Characterization includes X-ray diffraction (XRD), scanning electron microscopy (SEM), d_31_ piezoelectric coefficient, relative dielectric permittivity, and breakdown field measurements. Results illustrate the impacts of the studied parameters and demonstrate a fourfold increase in d_31_, compared to AlN, confirming the strong potential of Al_0.6_Sc_0.4_N for high-performance SMR transduction.

## 1. Introduction

Suspended microchannel resonators (SMRs) have emerged as promising tools for characterizing liquid reagents and measuring the mechanical properties of biological samples at the single-entity level [1,2,3,4,5,6]. Out of the numerous options for the motion transduction [5], piezoelectric transduction has been developed to offer a fast, linear, and low-power alternative [7]. To date, the two materials employed for this purpose are Lead Zirconate Titanate Pb [Zr_x_Ti_1−x_] O_3_ (PZT) [8] and AlN [9,10]. While the former exhibits higher transduction efficiency in miniaturized devices [11,12,13,14], the latter offers the advantages of being lead-free. This has driven the development of Pb-free alternatives with higher coupling than AlN through its doping or alloying [15,16,17,18,19,20].

It was in this context that AlScN has garnered significant interest, as adding scandium was shown to significantly enhance piezoelectric properties [21]. Growth temperature studies revealed varying trends [22], with an optimal d_33_ coefficient at 43% Sc content—approximately four times higher than that of AlN. Further increase in Sc concentration beyond 43% caused a sharp decline. Subsequent work has demonstrated that such decline can be surpassed by interface engineering through the introduction of strain [23] or a lattice-matched lutetium (Lu) buffer layer [24].

AlScN has thus been widely studied, both from the perspective of its material properties and its integration into devices. This includes analyses of the influence of the sputtering parameters on film properties [25,26], the effect of the Sc content on its crystal orientation [27], control of the residual stress and abnormally oriented grains (AOGs) in the films [28], in addition to investigations into its various characteristics, whether piezoelectric, mechanical, etching behavior, or thermal stability [29,30,31,32,33]. The material has also been employed in the development of several devices, namely, super-high-frequency acoustic resonators [34], CMOS-compatible high-frequency acoustic filters [35], MEMS-based infrared (IR) detectors [36], hybrid surface acoustic wave (SAW)/bulk acoustic wave (BAW) resonators [37], and lamb wave resonators with a scalable fabrication process [38].

This work investigates the use of Al_0.6_Sc_0.4_N thin films for SMR transduction, with selected sub-200 nm thicknesses tailored to our sensing application. Reducing the thickness of the piezoelectric layer effectively lowers the device’s effective mass and increases the electromechanical transduction efficiency, ultimately yielding a better device resolution [5,39]. However, excessive reduction in the piezoelectric film thickness can degrade the crystalline quality due to challenges in achieving sufficient (0002) lattice growth orientation during sputtering [33,40,41,42].

Previous work has investigated AlScN thin films with thicknesses ranging from 20 to 500 nm [42], aiming to characterize their dielectric and piezoelectric properties by examining Sc concentrations of 28%, 31%, and 36%. Characterization included the evaluation of rocking curves, the d_33_ coefficient, butterfly curves, capacitance, and loss tangent versus voltage. In the present work, we focus on films with 40% Sc content, studying the influence of fabrication and deposition parameters on film quality and assessing its suitability for SMR transduction.

To assess the influence of key process parameters on film quality, the piezoelectric stack is integrated into ls-SiN_x_ beam resonators. The impacts of the bottom electrode design, the photoresist removal method prior to deposition, and the deposition bias are examined. The selection of the studied parameters is deliberate, as it is building upon previous work that demonstrated the impact of deposition bias and electrode patterning on the film properties [33]. It additionally highlighted, along with a previous study [43], the critical role of optimizing photoresist removal, as residual contamination can significantly degrade the quality of subsequently deposited films. XRD, SEM, as well as the measurement of the d_31_ piezoelectric coefficient were employed for characterization. Additionally, the film’s relative dielectric permittivity and breakdown field were evaluated.

## 2. Materials and Methods

### 2.1. Film Deposition

For the deposition of the piezoelectric stack, comprising the bottom and top metal and AlScN films, we use a multi-chamber, single-target sputtering cluster, Spider600 (Pfeiffer Vaccum GmbH, Asslar, Germany). The tool is equipped with four process modules, each permitting gas lines of Ar and either N_2_ or O_2_ for reactive sputtering. Consequently, up to four different materials/alloys can be deposited in turn without exposure to the atmosphere. Metals are sputtered in DC mode, while nitrides (including AlN and AlScN) are sputtered using pulsed DC. The tool allows the substrate to heat up to 350 °C with the possibility of defining temperature profiles. Substrate bias can be applied via an RF generator at 13.56 MHz and is controlled through the setting of its DC power supply.

The deposition of the piezoelectric stack is carried out in two steps. First, the bottom electrode is deposited, consisting of an AlN adhesion layer followed by a Pt layer as the bottom metal. The two films are deposited consecutively without breaking the vacuum. The bottom electrode is then patterned, as detailed in the following subsection. Subsequently, the second deposition step is performed, which involves the deposition of the Al_0.6_Sc_0.4_N active piezoelectric layer and the top Pt layer, similarly without vacuum breaking.

For our process, the following three different targets are used: a Pt target with a purity of 99.95%, an Al target with a purity of 99.9995% for the reactive sputtering of AlN, and an Al-Sc cast target containing 40 at.%. Sc (52.6 wt.%) with a purity of 99.9% for the reactive sputtering of Al_0.6_Sc_0.4_N. The Al-Sc cast target was custom-made by Neyco (Vanves, France) upon request. Each target has a thickness of 6 mm and a diameter of 200 mm, and the target-to-wafer holder distance is 42.6 mm. Prior to deposition, two main steps are performed using a dummy wafer, as follows: First, 90 min substrate holder is heated. The step is executed through a ramp profile, heating to 300 °C. The heating step is followed by target conditioning. For Pt, this involves cleaning the target by sputtering onto the dummy wafer in a pure Ar atmosphere. For AlN and Al_0.6_Sc_0.4_N, the chamber is conditioned through a 20 min sputtering process using 50 SCCM of N_2_. The substrate is fixed during both steps.

The deposition recipe of each of the films includes a 5 min thermalization step, where the substrate is fixed and similarly heated to 300 °C. We begin with the sputtering of the bottom electrode layers; the AlN adhesion layer is deposited with 40 SCCM N_2_, and 10 SCCM Ar at 1500 W with no substrate bias, followed by Pt deposition at 15 SCCM Ar and 500 W. After patterning the bottom electrode, Al_0.6_Sc_0.4_N, the top Pt layers are deposited. The different layers are deposited at 300 °C. Building on the previous work [33] that investigated the effect of deposition bias from 2–8 W and identified 2 W as optimal when using 10–20 SCCM Ar and 30 SCCM N_2_, we explore whether a further reduction in the bias could enhance the piezoelectric film quality. Al_0.6_Sc_0.4_N is deposited at 1500 W using 30 SCCM N_2_ and 10 SCCM Ar, resulting in a pressure of ~4 × 10^−3^ mbar. Half of the wafers are deposited at 1 W substrate bias, and the other half at 0 W. The top Pt layer is then deposited using the same parameters as the bottom Pt, without breaking the vacuum.

The deposition rate of the Al_0.6_Sc_0.4_N with the set parameters is characterized to be 48 ± 2.4 nm/min. Accordingly, the deposition time is adjusted to 2 and 4 min to obtain film thicknesses of 100 nm or 200 nm, respectively. A further reduction in thickness leads to a lower breakdown voltage and an increased risk of pinhole formation.

### 2.2. Film Characterization

To characterize the crystallinity of our AlScN films, we use a D8 TXS X-ray diffractometer (Bruker AXS GmbH, Karlsruhe, Germany) equipped with a rotating anode source, an Eiger 500 2D, and a 2-bounce monochromator to perform both θ–2θ and rocking curve scans. Additionally, we examine the wafers with the SEM to detect the presence of AOGs and qualitatively compare them, evaluating the impact of the photoresist removal process on the quality of the piezoelectric film.

### 2.3. Device Fabrication

**Figure 1:**
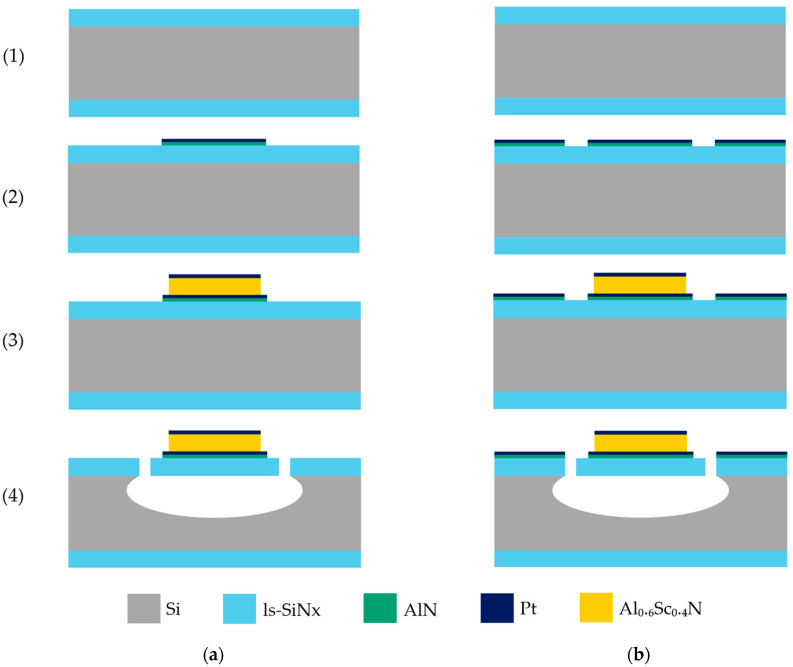
The simplified resonator beams fabrication process. (1) LPCVD of ls-SiN_x_ on a Si wafer, (2) deposition and patterning of the AlN adhesion layer and the Pt bottom electrode, (3) sputter deposition and patterning of the Al_0.6_Sc_0.4_N active layer and the Pt top electrode, (4) dry etching of the ls-SiNx and Si for the release of the resonator beams. There are two distinct designs for the bottom metal patterning. (**a**) shows the wafer with ~3.7% of bottom metal coverage; (**b**) shows the wafer with ~97.5% of bottom metal coverage.

The fabrication of a piezoelectrically transduced SMR is rather a lengthy and intricate process [5,9,39,44], which is why in this work, simpler 500 nm ls-SiN_x_ beam resonators with the piezoelectric stack are fabricated. We outline in the following section the details of the 3-mask fabrication process, whose schematic can also be found in Figure 1.

We begin by sputter-depositing the bottom electrode onto a 4-inch single-side polished Si <100> substrate with a 500 nm low-pressure chemical vapor deposition (LPCVD) ls-SiN_x_ film. Pt is selected for both the top and bottom electrodes for the following two key reasons: first, its <111> oriented nucleation promotes the growth of (0002) AlScN [45], and second, its chemical inertness and stability make it well-suited for our fabrication process and application [44]. To enhance the adhesion of the bottom Pt, a 25 nm AlN layer is deposited at 300 °C, and a 25 nm layer of the metal is then deposited at the same temperature and without breaking the vacuum.

Since most AlScN deposition studies use a non-patterned bottom electrode to ensure film quality and reproducibility by maintaining a vacuum, we aim to investigate how the bottom electrode patterning design affects the film’s properties. Two designs are tested, as follows:

- The first design features rectangular structures over the future resonator areas, connected to 400 µm × 400 µm pads, covering ~3.7% of the wafer’s area.
- The second design adds continuous metal across the wafer, separated by trenches for isolation, mimicking non-patterned coverage with ~97.5% metal coverage.

Photoresist coating is performed using an ACS200 GEN3 (SÜSS MicroTec, Garching Germany). After thermal dehydration at 135 °C, a 1 µm thick AZ^®^ ECI 3007 photoresist (MicroChemicals GmbH, Ulm, Germany) is dynamically dispensed at 1650 RPM and spun at 2050 RPM for uniformity, then baked at 100 °C for 90 s. Exposure is carried out using an MLA150 maskless aligner (Heidelberg Instruments Mikrotechnik GmbH, Heidelberg, Germany) with a 405 nm laser, followed by a post-exposure bake at 110 °C for 1 min. For the development, we implement a two-cycle puddle process (30 s each) with AZ^®^ 726 MIF developer (MicroChemicals GmbH, Ulm, Germany). A reflow step at 135 °C for 2 min follows to prevent fence formation during etching. Avoiding fences is critical to, in turn, avoid or minimize short-circuiting in our devices. Ion beam etching (IBE) is carried out using a Nexus IBE350 (Veeco Instruments Inc., Plainview, NY, USA), with an Ar ion beam current of 800 mA/cm^2^ and acceleration voltage of 500 V at −10° fixture angle.

Common photoresist removal methods include O_2_ plasma or wet stripping, but the chosen method can significantly impact AOG concentration in AlScN films [33]. This is likely due to ion beam-induced surface modifications that make the resist harder to strip, leading to a crust that impedes piezoelectric film growth [46]. To investigate this effect, we process two wafer batches. One batch undergoes dry O_2_ etching alone (5 min, 400 SCCM O_2_, 600 W), which we refer to as the dry removal batch. For the other, a combined three-step method is implemented, with O_2_ plasma pre-treatment (1 min, 400 SCCM, 600 W), two 5 min baths in N-methyl-2-pyrrolidone (Remover 1165, Dow Inc., Midland, MI, USA) at 70 °C, and finally O_2_ plasma (3 min, 200 SCCM, 200 W). This batch of wafers is later referred to as the dry + wet removal batch.

Following the patterning of the bottom electrode, the Al_0.6_Sc_0.4_N active layer is deposited using the previously described parameters, followed by a 25 nm top Pt layer, all without breaking vacuum. Photolithography and ion beam etching are then used to pattern the top Pt electrode using the same designs across all wafers. The same mask and IBE recipe were used to etch the AlScN layer.

Care must be taken to avoid over-etching during IBE, as it leads to the undesired etching of the relatively thin 25 nm bottom Pt layer. Given that the process is purely physical, it offers no selectivity between AlScN and Pt. Therefore, the piezoelectric film etching is adjusted to remove most of the film thickness using IBE, with the final etching step completed through wet etching The IBE process is thus calibrated to leave an approximate 30 nm thickness of AlScN unetched. Subsequently, the resist is stripped using our standard O_2_ plasma. The wafer is then immersed in an AZ^®^ 400k developer (MicroChemicals GmbH, Ulm, Germany), a potassium-based buffered solution known to etch Al compounds.

A final lithography step is carried out to define the resonator beams. The ACS200 GEN3 is used for surface preparation with HMDS vapor priming, followed by coating with a 4 µm thick AZ^®^ 10XT-20 photoresist (MicroChemicals GmbH, Ulm, Germany). The resist is dynamically dispensed at 1050 RPM and spun at the same speed for uniform coverage. A soft bake is performed at 110 °C for 210 s in minimum proximity. Exposure is carried out using the MLA150 maskless aligner with a 405 nm laser source. Development is completed on the ACS200 GEN3 using AZ 400k Developer (MicroChemicals GmbH, Ulm, Germany) diluted 1:3.5 with water. The development process includes a 12 s spray, followed by three 60 s puddle cycles.

To release our beams, nitride etching is first performed using an APS Dielectric Etcher (SPTS Technologies Ltd., Newport, UK) with a C_4_F_8_/H_2_/He chemistry. Silicon etching is then carried out on a Rapier DSE (SPTS Technologies Ltd., Newport, UK), starting with a Bosch deep reactive ion etching (DRIE) process, followed by isotropic SF_6_ etching to ensure full release. Finally, the photoresist is stripped using dry O_2_ plasma to prevent stiction and breaking of beams during drying.

**Figure 2:**
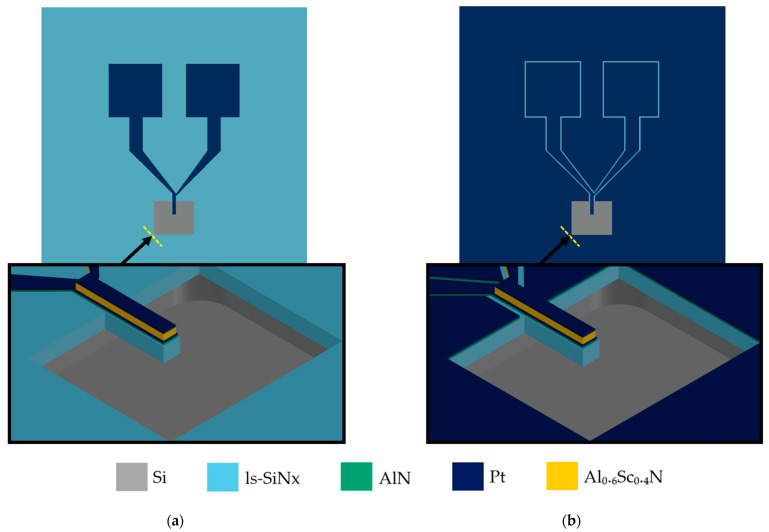
A schematic showing the top and side views of the fabricated devices, where (**a**) shows a device on the 3.7% bottom metal coverage wafer; (**b**) shows a device on the 97.5% bottom metal coverage wafer.

The fabricated suspended beams have a width of 25 µm, with the following three different lengths implemented: 50, 100, and 150 µm. As detailed in this section, the layer stack consists of 500 nm ls-SiN_x_, a 25 nm AlN + 25 nm Pt bottom electrode, the Al_0.6_Sc_0.4_N piezoelectric layer (either 100 or 200 nm thick), and a 25 nm Pt top electrode. Since only piezoelectric actuation is required for this study, with detection performed optically as described in the following section, a single top electrode was defined. The fabricated devices are depicted in Figure 2. Designed test structures are also implemented in the design to serve the measurement of the relative dielectric permittivity, as explained in the next section.

### 2.4. Measurement of the d_31_ Coefficient

**Figure 3:**
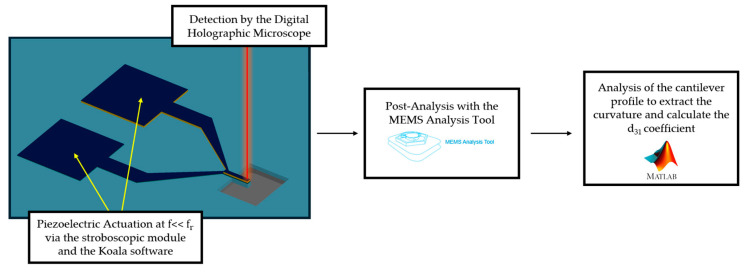
A schematic of the measurement and analysis setup. The cantilevers are actuated by the stroboscopic module, and their out-of-plane movement is captured by the DHM. Data are analyzed by the MEMS analysis tool, and the profile is fitted by a custom script to calculate the d_31_ coefficient.

To characterize the piezoelectric coefficient of our film, we focus on the measurement of the d_31_ coefficient, which is the critical parameter for SMR transduction (and more generally, any flexural resonator). We conduct dynamic mode displacement measurements where the resonators are actuated off-resonance, and their displacement profile is evaluated. This methodology was chosen to eliminate the effect of stress variation and initial bending of the beams. The measurement setup, shown in Figure 3, employs a Reflection Digital Holographic Microscope DHM^®^-R2100 (Lyncée Tec SA, Ecublens, Switzerland) equipped with a stroboscopic module and the MEMS AnalysisTool 7.3 software (Lyncée Tec SA, Ecublens, Switzerland) for post-processing. Actuation is carried out via the stroboscopic module, controlled by the Koala 8.5 Acquisition and Analysis software (Lyncée Tec SA, Ecublens, Switzerland), enabling capture of the out-of-plane resonator movement [47,48]. The acquired data are then processed with the MEMS Analysis software to extract displacement profiles.

The displacement information is later fed into a custom MATLAB (R2025a) script that fits the dynamic profile to a parabola and calculates the d_31_ coefficient based on the following equation [49,50]:

(1) $$d_{31}=\frac{\frac{1}{R}\sum_{i}E_{i}A_{i}(\frac{t_{i}^{2}}{12}+Z_{i})}{E_{p}Z_{p}w_{p}V},$$

where R represents the radius of curvature of the fitted profile curve, $E_{i}$ denotes the Young’s modulus of the layer i, $A_{i}$ is its cross-sectional area (calculated as the product of its width $w_{i}$ and its thickness $t_{i}$), and $Z_{i}$ is the position of its center with respect to the neutral axis. The subscript p refers to the piezoelectric layer where $E_{p}$, $Z_{p}$, and $w_{p}$ represent its Young’s modulus, the position of its center to the neutral axis, and its width, respectively. V is the applied actuation voltage.

Cantilevers with lengths of 50, 100, and 150 µm are measured. For each wafer, three devices of the same length are examined at three different actuation voltages, with five measurements taken for each. This results in a total of 135 measurements per wafer, and the data are then analyzed individually. The devices are also selected to span different parts of the wafer, thus accounting for any variability in the film. This protocol is followed to ensure the reproducibility and consistency of the measurements.

### 2.5. Measurement of the Breakdown Field and Dielectric Constant

Further electrical characterization of the piezoelectric films is performed using a TF 3000 measurement system (aixACCT Systems GmbH, Aachen, Germany). We start by measuring the breakdown voltage by probing the top and bottom electrodes and applying a voltage range whilst plotting the I–V curve to determine the resistance. The voltage range is incrementally increased until breakdown occurs, indicated by a relatively small resistance value between the top and bottom, due to the creation of a conductive path between the electrodes. The corresponding voltage is recorded.

We then proceed to measure predesigned rectangular test structures of the piezoelectric stack. By connecting the top and bottom metal layers, we calculate the capacitance of the structure via measurement of the current and the instantaneous voltage rate of change. Given the known area of the test structures—deliberately designed large enough to render the parasitic effects negligible—and the piezoelectric film thickness, the dielectric constant can hence be extracted.

## 3. Results

To evaluate the chosen parameters on the piezoelectric film quality, we fabricate two wafer batches—one with the bottom metal layer covering 97.5% of the wafer area and another with only 3.7% metal coverage. In the 97.5% coverage batch, we vary the resist removal method, where we use dry removal only on some wafers and dry + wet removal on others. For the 3.7% coverage batch, we limit the resist cleaning method to dry removal to reduce the number of variables. For each combination of bottom electrode design and resist removal method, we study two different film thicknesses—100 nm and 200 nm. For each thickness, we also vary the deposition bias examining values of 0 W and 1 W. The arrangements of the fabricated wafers are summarized in Table 1.

### 3.1. XRD Analysis

**Figure 4:**
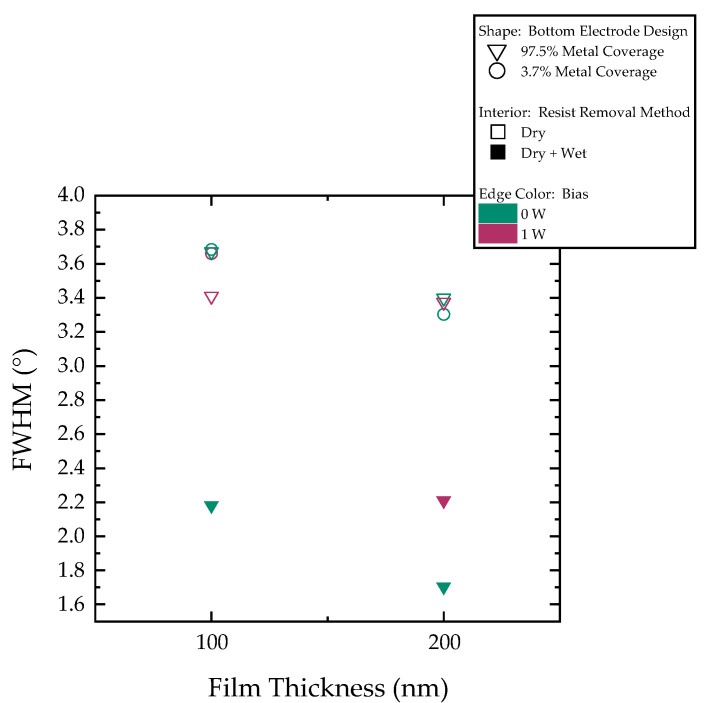
Rocking curve FWHM plot. Point shape categorizes wafers based on their bottom electrode design, whether 97.5% or 3.7% metal coverage. Point fill indicates resist removal method—hollow for dry photoresist removal only, solid for dry + wet removal. Edge color denotes deposition bias power.

We analyze the rocking curve results of the two batches. Our goal is to assess the influence of the studied parameters on the full width half maximum (FWHM) value of the rocking curve. In principle, the FWHM value is an indicator of crystalline orientation as a highly oriented crystal will result in a sharp peak and hence a smaller FHWM value. Additionally, we aim to examine whether the results vary based on film thickness. The obtained values are presented in Figure 4.

The resist photoresist cleaning method is observed to have a significant effect on film growth and, consequently, its crystalline quality. Wafers subjected to dry + wet removal exhibit narrower peaks, reflected by smaller FWHM values. Moreover, this cleaning step appears to affect the film’s sensitivity to the other process parameters as discussed in the next section in further detail. It can also be seen that, although the influence of film thickness is less pronounced in wafers that undergo only dry resist removal, films with thickness of 200 nm generally exhibited superior crystalline quality. Notably, challenges are encountered in obtaining a clear rocking curve peak for the wafer from the second batch with a 100 nm film deposited at a 1 W bias, despite a satisfactory θ–2θ scan.

### 3.2. SEM Examination

**Figure 5:**
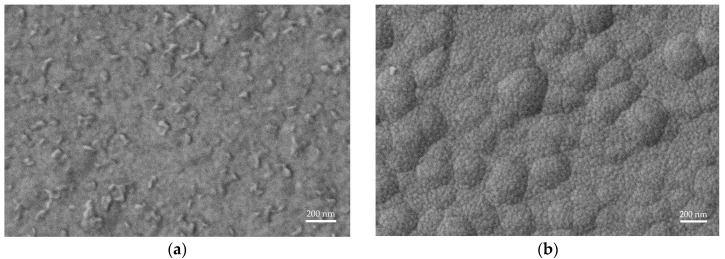
Comparison of wafer surface SEM images showing the impact of photoresist removal on AOG formation. (**a**) Dry removal results in a high density of AOGs. (**b**) Dry + wet removal eliminates AOGs, yielding a clean surface. SEM examination also reveals differences in the underlying ls-SiN_x_ grain sizes dependent on the LPCVD run.

We proceed to carry out an SEM examination where all wafers are thoroughly examined to assess the presence of AOGs qualitatively. The examination leads us to find a prominent distinction based on the resist removal method as reported in Figure 5, where the wafers subjected to dry photoresist removal show a high presence of AOGs when compared to the wafers where dry + wet removal is implemented in the fabrication process.

The examination also reveals the presence of two distinct grain sizes on the surface. The smaller grains originate from the sputtered layers, namely AlScN and Pt, while the larger and smoother, hill-like grains stem from the underlying ls-SiN_x_. This feature of the LPCVD layers has been previously observed and strongly depends on the furnace’s condition, leading to variations in surface roughness between runs.

### 3.3. Measurement of the d_31_ Coefficient, the Dielectric Relative Permittivity, and the Breakdown Field

**Figure 6:**
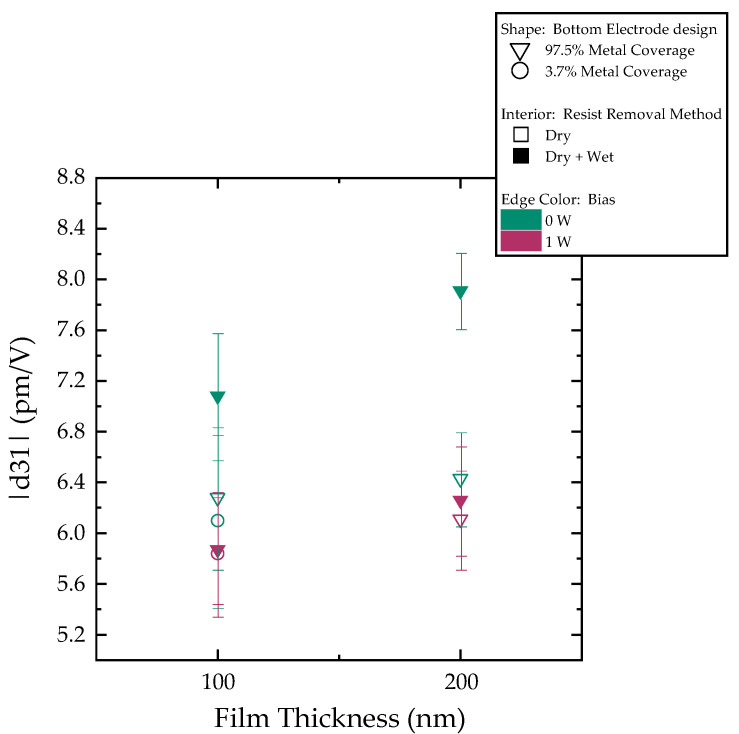
Measured d_31_ coefficients. The point shape differentiates wafer types by bottom metal design, the point fill indicates the resist removal method, and the point edge color indicates deposition bias.

Finally, measurements of the d_31_ coefficient are conducted. In Figure 6, we present the results for the different wafers. The coefficient values ranged between −5.3 and −8.2 pm/V. The resist removal method strongly influences the results. With dry + wet removal and 97.5% bottom metal coverage, higher d_31_ is achieved with 0 W bias deposition.

Additionally, a characterization of the dielectric constant and the breakdown voltage was carried out. We find the relative dielectric permittivity of our films to be 30±3. The breakdown voltage is also characterized, and assuming a uniform electric field, we can calculate the breakdown field value from the film thicknesses to be around 2.2±0.5 MV/cm. When measuring the different values of the relative dielectric permittivity and the breakdown voltage, it was observed that the dispersion within one wafer is larger than the dispersion from wafer to wafer. This indicates a negligible or no influence of the deposition and fabrication parameters.

## 4. Discussion

Examination of the reported XRD results reveals a clear difference in the FWHM values between the wafers according to their resist removal process. The ones that went through only dry removal show FWHM values ranging from 3.1° to 3.7°. In comparison, the wafers that were subjected to a dry + wet removal exhibit significantly narrower peaks with values ranging from 1.7° to 2.2°, thus indicating improved crystalline quality and closer alignment with previously reported values in the literature on other substrates [33,42]. This demonstrates the influence of the photoresist cleaning method, prior to piezoelectric deposition, on film growth.

SEM analysis confirms our observations, as it reveals the presence of AOGs across all wafers on which dry removal is applied, higher concentration in the 200 nm films, implying deposition-related defects. In contrast, AOG occurrence is nearly absent when dry + wet removal is implemented, highlighting the critical role of the photoresist stripping step in film growth. When the photoresist is subjected to an ion beam, its upper layer is chemically modified to form what can be described as a burnt layer or a ‘crust’ [46]. This crust is resistant to oxygen stripping and hence is not fully removed when only dry photoresist removal is used. The photoresist residues then hinder the growth of the subsequent piezoelectric layer during sputtering, triggering the formation of AOGs. In contrast, when dry + wet removal is employed, the photoresist residues that persist through O_2_ stripping are lifted off during wet photoresist stripping. The resulting clean surface of the bottom metal layer promotes the growth of the AlScN film with no AOGs.

The following two distinct grain sizes are also identified via the SEM examination: a small grain size originating from the sputtered AlScN and Pt layers and a larger grain size with hill-like morphology stemming from the underlying LPCVD ls-SiN_x_. This feature of the LPCVD layer strongly depends on the state of the furnace, resulting in surface roughness variations between the deposition runs, as seen in the examined wafers.

Closer examination of the XRD results of the wafers with dry resist removal shows no clear influence of the bottom electrode patterning design or the deposition bias, as all wafers exhibit similar FWHM values. In addition, no significant difference is observed between 100 nm and 200 nm films with the dry resist removal. Conversely, the wafers subjected to dry + wet removal show a more pronounced thickness effect; for films deposited with 0 W bias, the FWHM decreases from 2.2° (100 nm) to 1.7° (200 nm), aligning with prior findings where the quality plateaus beyond 200 nm [33]. Although data from the 100 nm film deposited at 1 W bias would have helped validate this trend, it was unobtainable, and we relied on d_31_ measurements to complement the analysis.

Consistent with the XRD and SEM findings, the d_31_ measurements reveal a clear distinction between the two wafer cleaning methods. The influence of the bottom electrode patterning design, the film thickness, and deposition bias is insignificant when dry removal is implemented, falling within the measurement error. The d_31_ values average around −6.1 ± 0.7 pm/V. When dry + wet removal is used, however, wafers exhibit improved values, underlining the impact of the photoresist cleaning step. Notably, films deposited after dry + wet resist removal with 0 W bias perform better, with a more pronounced difference between 100 nm and 200 nm thickness. Under optimal conditions of dry + wet cleaning and 0 W bias, devices with the 100 nm thick piezoelectric film exhibit a piezoelectric coefficient of −7 ± 0.5 pm/V. Under the same conditions, the 200 nm films achieve a fourfold increase in d_31_ (−8 ± 0.3 pm/V) when compared to AlN’s −2 pm/V. The measured piezoelectric coefficient values were found to be only moderately smaller than previously reported values for thicker films [33,51], thus highlighting the material’s strong potential even at relatively small thicknesses.

Unlike the piezoelectric coefficient, the relative dielectric permittivity and the breakdown electric field are unaffected by the variation of either the fabrication or deposition parameters. Additionally, the extracted relative dielectric permittivity aligns with previously reported values [33,52,53,54]. As for the obtained breakdown field, despite being lower than reported values for 36% Sc concentration [55], the measured breakdown voltages are well above the low actuation levels used in SMRs, eliminating concerns of dielectric failure.

## 5. Conclusions

This study investigates sub-200 nm Al_0.6_Sc_0.4_N thin films for SMRs’ piezoelectric transduction, benchmarking their performance against AlN. Integrated into ls-SiN_x_ cantilever resonators, the effects of bottom electrode patterning design, resist removal, and deposition bias on film quality are examined. A combined electrical and structural characterization approach reveals key material properties, namely crystallinity, the d_31_ piezoelectric coefficient, the relative dielectric permittivity, and the breakdown field. The photoresist removal method has been proven critical to film quality, as the impact of photoresist residues suppresses the effect of other fabrication and deposition parameters. The highest d_31_ coefficient value obtained demonstrated a fourfold increase compared to undoped AlN, despite reduced film thickness. Such results confirm AlScN’s strong potential for high-performance SMRs. The studied parameters are shown not to affect the relative permittivity and breakdown field of the piezoelectric films. The relative dielectric permittivity aligns well with the literature values. The measured breakdown field, while slightly lower than reported values for thicker films, remains adequate for the functional parameters of SMRs.

## Figures and Tables

**Table 1:** A summary of the wafer arrangements for the study of the different parameters.

Bottom Electrode Design	Resist Removal Method	Film Thickness	Bias
97.5% Metal Coverage	Dry	100 nm	0 W
			1 W
		200 nm	0 W
			1 W
	Dry + Wet	100 nm	0 W
			1 W
		200 nm	0 W
			1 W
3.7% Metal Coverage	Dry	100 nm	0 W
			1 W
		200 nm	0 W
			1 W

## Data Availability

The original contributions presented in this study are included in the article. Further inquiries can be directed to the corresponding author.

## Abbreviations

The following abbreviations are used in this manuscript:

SMR	Suspended microchannel resonator
Ls-SiN_x_	Low-stress silicon nitride
XRD	X-ray diffraction
SEM	Scanning electron microscopy
PZT	Lead Zirconate Titanate Pb [Zr_x_Ti_1−x_] O_3_
AOGs	Abnormally oriented grains
IR	Infrared
SAW	Surface acoustic waves
BAW	Bulk acoustic waves
LPCVD	Low-pressure chemical vapor deposition
IBE	Ion beam etching
DRIE	Deep reactive ion etching
DHM	Digital holographic microscope
FWHM	Full-width half-maximum

## References

[1] Lee J., Chunara R., Shen W., Payer K., Babcock K., Burg T.P., Manalis S.R. (2011). Suspended Microchannel Resonators with Piezoresistive Sensors. Lab Chip.

[2] Calleja M., Kosaka P.M., Paulo Á.S., Tamayo J. (2012). Challenges for Nanomechanical Sensors in Biological Detection. Nanoscale.

[3] Khan M.F., Kim S., Lee D., Schmid S., Boisen A., Thundat T. (2014). Nanomechanical Identification of Liquid Reagents in a Microfluidic Channel. Lab Chip.

[4] Cermak N., Olcum S., Delgado F.F., Wasserman S.C., Payer K.R., A Murakami M., Knudsen S.M., Kimmerling R.J., Stevens M.M., Kikuchi Y. (2016). High-Throughput Measurement of Single-Cell Growth Rates Using Serial Microfluidic Mass Sensor Arrays. Nat. Biotechnol..

[5] De Pastina A., Villanueva L.G. (2020). Suspended Micro/Nano Channel Resonators: A Review. J. Micromech. Microeng..

[6] Martín-Pérez A., Ramos D., Tamayo J., Calleja M. (2021). Nanomechanical Molecular Mass Sensing Using Suspended Microchannel Resonators. Sensors.

[7] Cai X., Wang Y., Cao Y., Yang W., Xia T., Li W. (2024). Flexural-Mode Piezoelectric Resonators: Structure, Performance, and Emerging Applications in Physical Sensing Technology, Micropower Systems, and Biomedicine. Sensors.

[8] Groenesteijn J., de Boer M.J., Lötters J.C., Wiegerink R.J. (2017). A Versatile Technology Platform for Microfluidic Handling Systems, Part I: Fabrication and Functionalization. Microfluid. Nanofluid..

[9] De Pastina A., Maillard D., Villanueva L.G. (2018). Fabrication of Suspended Microchannel Resonators with Integrated Piezoelectric Transduction. Microelectron. Eng..

[10] Maillard D., De Pastina A., Abazari A.M., Villanueva L.G. (2021). Avoiding Transduction-Induced Heating in Suspended Microchannel Resonators Using Piezoelectricity. Microsyst. Nanoeng..

[11] Howell K.M., De Pastina A., Lozzi A., Larsen T., Faizan M., Villanueva L.G. Piezoelectric Nanoelectromechanical Systems. Proceedings of the 2017 19th International Conference on Solid-State Sensors, Actuators and Microsystems (TRANSDUCERS).

[12] Polcawich R.G., Pulskamp J.S., Bhugra H., Piazza G. (2017). Lead Zirconate Titanate (PZT) for M/NEMS. Piezoelectric MEMS Resonators.

[13] Saya D., Dezest D., Welsh A.J., Mathieu F., Thomas O., Leïchlé T., Trolier-McKinstry S., Nicu L. (2020). Piezoelectric Nanoelectromechanical Systems Integrating Microcontact Printed Lead Zirconate Titanate Films. J. Micromech. Microeng..

[14] Liu J., Tan H., Zhou X., Ma W., Wang C., Tran N.-M.-A., Lu W., Chen F., Wang J., Zhang H. (2025). Piezoelectric Thin Films and Their Applications in MEMS: A Review. J. Appl. Phys..

[15] Yokoyama T., Iwazaki Y., Onda Y., Nishihara T., Sasajima Y., Ueda M. (2015). Highly Piezoelectric Co-Doped AlN Thin Films for Wideband FBAR Applications. IEEE Trans. Ultrason. Ferroelectr. Freq. Control.

[16] Manna S., Brennecka G.L., Stevanović V., Ciobanu C.V. (2017). Tuning the Piezoelectric and Mechanical Properties of the AlN System via Alloying with YN and BN. J. Appl. Phys..

[17] Manna S., Talley K.R., Gorai P., Mangum J., Zakutayev A., Brennecka G.L., Stevanović V., Ciobanu C.V. (2018). Enhanced Piezoelectric Response of AlN via CrN Alloying. Phys. Rev. Appl..

[18] Hirata K., Mori Y., Yamada H., Uehara M., Anggraini S.A., Akiyama M. (2021). Significant Enhancement of Piezoelectric Response in AlN by Yb Addition. Materials.

[19] Yu X., Zhu L., Li X., Zhao J., Wu T., Yu W., Li W. (2023). Doping Engineering for Optimizing Piezoelectric and Elastic Performance of AlN. Materials.

[20] Startt J., Quazi M., Sharma P., Vazquez I., Poudyal A., Jackson N., Dingreville R. (2023). Unlocking AlN Piezoelectric Performance with Earth-Abundant Dopants. Adv. Electron. Mater..

[21] Akiyama M., Kamohara T., Kano K., Teshigahara A., Takeuchi Y., Kawahara N. (2009). Enhancement of Piezoelectric Response in Scandium Aluminum Nitride Alloy Thin Films Prepared by Dual Reactive Cosputtering. Adv. Mater..

[22] Akiyama M., Kano K., Teshigahara A. (2009). Influence of Growth Temperature and Scandium Concentration on Piezoelectric Response of Scandium Aluminum Nitride Alloy Thin Films. Appl. Phys. Lett..

[23] Hirata K., Shobu K., Yamada H., Uehara M., Anggraini S.A., Akiyama M. (2020). Thermodynamic Assessment of the Al–Sc–N Ternary System and Phase-Separated Region of the Strained Wurtzite Phase. J. Eur. Ceram. Soc..

[24] Hirata K., Niitsu K., Anggraini S.A., Kageura T., Uehara M., Yamada H., Akiyama M. (2025). Enhancing the Piezoelectric Performance of Nitride Thin Films through Interfacial Engineering. Mater. Today.

[25] Umeda K., Kawai H., Honda A., Akiyama M., Kato T., Fukura T. Piezoelectric Properties of ScAlN Thin Films for Piezo-MEMS Devices. Proceedings of the 2013 IEEE 26th International Conference on Micro Electro Mechanical Systems (MEMS).

[26] Lu Y., Reusch M., Kurz N., Ding A., Christoph T., Kirste L., Lebedev V., Žukauskaitė A. (2018). Surface Morphology and Microstructure of Pulsed DC Magnetron Sputtered Piezoelectric AlN and AlScN Thin Films. Phys. Status Solidi.

[27] Mertin S., Heinz B., Rattunde O., Christmann G., Dubois M.-A., Nicolay S., Muralt P. (2018). Piezoelectric and Structural Properties of C-Axis Textured Aluminium Scandium Nitride Thin Films up to High Scandium Content. Surf. Coat. Technol..

[28] Beaucejour R., Roebisch V., Kochhar A., Moe C.G., Hodge M.D., Olsson R.H. (2022). Controlling Residual Stress and Suppression of Anomalous Grains in Aluminum Scandium Nitride Films Grown Directly on Silicon. J. Microelectromech. Syst..

[29] Matloub R., Hadad M., Murait P. Piezoelectric Coefficients of AlScN Thin Films in Comparison. Proceedings of the 2016 IEEE International Frequency Control Symposium (IFCS).

[30] Lu Y., Reusch M., Kurz N., Ding A., Christoph T., Prescher M., Kirste L., Ambacher O., Žukauskaitė A. (2018). Elastic Modulus and Coefficient of Thermal Expansion of Piezoelectric Al1−xScxN (up to x = 0.41) Thin Films. APL Mater..

[31] Luo Z., Shao S., Wu T. (2021). Characterization of AlN and AlScN Film ICP Etching for Micro/Nano Fabrication. Microelectron. Eng..

[32] Österlund E., Ross G., Caro M.A., Paulasto-Kröckel M., Hollmann A., Klaus M., Meixner M., Genzel C., Koppinen P., Pensala T. (2021). Stability and Residual Stresses of Sputtered Wurtzite AlScN Thin Films. Phys. Rev. Mater..

[33] Liffredo M., Xu N., Stettler S., Peretti F., Villanueva L.G. (2024). Piezoelectric and Elastic Properties of Al0.60Sc0.40N Thin Films Deposited on Patterned Metal Electrodes. J. Vac. Sci. Technol. A.

[34] Park M., Hao Z., Dargis R., Clark A., Ansari A. (2020). Epitaxial Aluminum Scandium Nitride Super High Frequency Acoustic Resonators. J. Microelectromech. Syst..

[35] Giribaldi G., Colombo L., Rinaldi M. (2023). 6-20 GHz 30% ScAlN Lateral Field-Excited Cross-Sectional Lamé Mode Resonators for Future Mobile RF Front Ends. IEEE Trans. Ultrason. Ferroelectr. Freq. Control.

[36] Hussein H.M.E., Ayed F.B., Venditti A., Simeoni P., Qian Z., Cassella C., Rinaldi M. (2024). Parametric Frequency Comb Generator Based AlScN MEMS IR Detector for Low-Power and High-Performance Applications. Proc. SPIE.

[37] Liffredo M., Stettler S., Peretti F., Villanueva L.G. (2024). Rayleigh Wave Suppression in Al^0.6^Sc^0.4^N-on-SiC Resonators. arXiv.

[38] Liffredo M., Peretti F., Xu N., Stettler S., Villanueva L.G. (2024). More-than-Moore Microacoustics: A Scalable Fabrication Process for Suspended Lamb Wave Resonators. arXiv.

[39] De Pastina A. (2018). PZE-Transduced Suspended Microchannel Resonators for Sensing Applications. Ph.D. Thesis.

[40] Martin F., Muralt P., Dubois M.-A., Pezous A. (2004). Thickness Dependence of the Properties of Highly C-Axis Textured AlN Thin Films. J. Vac. Sci. Technol. A.

[41] Howell K.M., Bashir W., De Pastina A., Matloub R., Muralt P., Villanueva L.G. (2019). Effect of AlN Seed Layer on Crystallographic Characterization of Piezoelectric AlN. J. Vac. Sci. Technol. A.

[42] Pirro M., Herrera B., Assylbekova M., Giribaldi G., Colombo L., Rinaldi M. Characterization of Dielectric and Piezoelectric Properties of Ferroelectric AlScN Thin Films. Proceedings of the 2021 IEEE 34th International Conference on Micro Electro Mechanical Systems (MEMS).

[43] Yang Z., Wang Q., Qi L., Wang X., Li K., Chen D., Li C., Zou H. (2019). Double-layer Resist Method to Improve Descum Result When Removing Negative Photoresist. Micro Nano Lett..

[44] Maillard D. (2022). Piezoelectric Suspended Microchannel Resonators. Ph.D. Thesis.

[45] Fichtner S., Wolff N., Krishnamurthy G., Petraru A., Bohse S., Lofink F., Chemnitz S., Kohlstedt H., Kienle L., Wagner B. (2017). Identifying and Overcoming the Interface Originating C-Axis Instability in Highly Sc Enhanced AlN for Piezoelectric Micro-Electromechanical Systems. J. Appl. Phys..

[46] Hirose K., Shimada H., Shimomura S., Onodera M., Ohmi T. (1994). Ion-Implanted Photoresist and Damage-Free Stripping. J. Electrochem. Soc..

[47] Bhaskar U.K., Banerjee N., Abdollahi A., Solanas E., Rijnders G., Catalan G. (2016). Flexoelectric MEMS: Towards an Electromechanical Strain Diode. Nanoscale.

[48] Emery Y., Colomb T., Cuche E. (2021). Metrology Applications Using Off-Axis Digital Holography Microscopy. J. Phys. Photonics.

[49] Schmid S., Villanueva L.G., Roukes M.L. (2023). Fundamentals of Nanomechanical Resonators.

[50] Dekkers M., Boschker H., Van Zalk M., Nguyen M., Nazeer H., Houwman E., Rijnders G. (2013). The Significance of the Piezoelectric Coefficient D_31,eff_ Determined from Cantilever Structures. J. Micromech. Microeng..

[51] Akiyama M., Umeda K., Honda A., Nagase T. (2013). Influence of Scandium Concentration on Power Generation Figure of Merit of Scandium Aluminum Nitride Thin Films. Appl. Phys. Lett..

[52] Wang P., Wang D., Mondal S., Hu M., Liu J., Mi Z. (2023). Dawn of Nitride Ferroelectric Semiconductors: From Materials to Devices. Semicond. Sci. Technol..

[53] Chen J., Zhang J., Fan Z., Yu P. (2025). Microstructure and Electrical Properties of Scandium-Doped Aluminum Nitride Thin Film. Coatings.

[54] Kreutzer T.-N., Ghori M.Z., Islam M.R., Lofink F., Stoppel F., Müller-Groeling A., Fichtner S. (2025). Wafer Scale Reactive Sputtering of Highly Oriented and Ferroelectric Al^0.6^Sc^0.4^N from 300 Mm AlSc Targets. J. Micromech. Microeng..

[55] Zheng J.X., Wang D., Musavigharavi P., Fiagbenu M.M.A., Jariwala D., Stach E.A., Olsson R.H. (2021). Electrical Breakdown Strength Enhancement in Aluminum Scandium Nitride through a Compositionally Modulated Periodic Multilayer Structure. J. Appl. Phys..

